# Comparative Effects of 4% Articaine and 2% Lignocaine on Salivary Biomarkers of Pain and Stress in Surgical Extraction of Impacted Mandibular Third Molars: A Prospective Observational Study

**DOI:** 10.7759/cureus.94575

**Published:** 2025-10-14

**Authors:** Mohd Younis Bhat, Shahid Farooq, Nitika Mittal, Nayan Gupta, Neeta S Padmawar, Faisal Noor Ahmad, Manish Sharma, Reshma Hammannavar

**Affiliations:** 1 Oral and Maxillofacial Surgery, Government Dental College and Hospital, Srinagar, IND; 2 Dentistry, Government Medical College, Kathua, IND; 3 Anesthesia, Sarojini Naidu Medical College, Agra, IND; 4 Oral and Maxillofacial Surgery, Kothiwal Dental College and Research Centre, Moradabad, IND; 5 Pedodontics and Preventive Dentistry, Rural Dental College, Pravara Institute of Medical Sciences (Deemed to be University), Loni, IND; 6 Public Health Dentistry, School of Dental Sciences, Sharda University, Greater Noida, IND; 7 Oral Pathology, Jawahar Medical Foundation's Annasaheb Chudaman Patil Memorial Dental College, Dhule, IND; 8 Oral Surgery, Jawahar Medical Foundation's Annasaheb Chudaman Patil Memorial Dental College, Dhule, IND

**Keywords:** biomarkers, impacted, mandibular, pain, stress, third molar

## Abstract

Introduction: Surgical extraction of impacted mandibular third molars is a common dental procedure associated with significant pain and stress, which can be modulated by local anesthetics. This study aimed to assess the influence of 2% lignocaine versus 4% articaine on salivary biomarkers of pain and stress in patients undergoing surgical extraction of impacted mandibular third molars. The specific objective was to measure and compare the baseline and postoperative levels of cortisol and salivary alpha-amylase (sAA), along with the pain and anxiety scores, between the two anesthetic groups.

Materials and Methods: This prospective observational study was conducted at the Department of Oral and Maxillofacial Surgery between January and December 2022. 40 patients (aged 18-40 years, American Society of Anesthesiologists (ASA) I/II) with mesioangular impacted third molars were allocated to receive 2% lignocaine (n = 20) or 4% articaine (n = 20) based on clinical practice. Baseline pain (Visual Analog Scale (VAS) <10 mm), anxiety (Modified Dental Anxiety Scale (MDAS) 5-14), and unstimulated saliva (5 mL, 8-10 AM) were assessed. Saliva was collected at baseline (T0), 1-h post injection (T1), and 24-h post extraction (T24). The cortisol (enzyme-linked immunosorbent assay) and sAA (kinetic colorimetric assay) levels were measured in salivary samples. Pain was assessed using the VAS at same time intervals. Surgical complexity was standardized and data were analyzed using independent t-tests, repeated-measures (RM) analysis of variance (ANOVA), and two-way ANOVA (p < 0.05).

Results: Baseline characteristics were comparable (p > 0.05). RM ANOVA showed significant changes over time in pain, cortisol, and sAA levels in both groups (p = 0.001). Two-way ANOVA revealed significant group effects for cortisol (p = 0.008) and sAA (p = 0.021), and interactions (cortisol: p = 0.038; sAA: p = 0.047). 4% articaine showed lower pain (16.25 ± 5.72 vs. 20.5 ± 3.68, p = 0.008), cortisol (0.15 ± 0.06 µg/dL vs. 0.24 ± 0.13 µg/dL, p = 0.009), and sAA reductions (T24-T0) (22.63 ± 8.6 units/mL vs. 45.03 ± 16.01 units/mL, p = 0.001) compared to 2% lignocaine.

Conclusion: Compared to 2% lignocaine, 4% articaine demonstrated superior modulation of pain and stress biomarkers, supporting its preferential use in impacted mandibular third molar extraction.

## Introduction

Surgical extraction of impacted third molars is one of the most common procedures in oral and maxillofacial surgery and is often associated with significant perioperative pain, anxiety, and physiological stress responses [1]. Impacted third molars, particularly mandibular molars, affect a substantial portion of the population, with global prevalence rates ranging from 9.5% to 68%, leading to complications such as pericoronitis, caries, and cyst formation if left untreated [2]. These surgeries can evoke acute stress, manifesting as elevated heart rate, blood pressure, and cortisol levels, which may exacerbate postoperative recovery challenges including swelling, trismus, and delayed healing [3]. Pain management is crucial because inadequate control can lead to chronic pain syndromes or increased opioid reliance, highlighting the need for optimized anesthetic strategies.

Local anesthetics play a pivotal role in mitigating intraoperative and postoperative discomfort during third molar extraction. Commonly used agents include lidocaine, articaine, bupivacaine, and mepivacaine, each differing in onset time, duration of action, potency, and potential side effects [4]. For instance, articaine offers rapid onset and superior diffusion through the bone, making it effective for mandibular blocks, whereas bupivacaine provides prolonged analgesia suitable for extended postoperative pain relief [5]. Comparative studies have shown variations in efficacy; articaine often requires fewer reinjections and achieves faster anesthesia than lidocaine or bupivacaine, potentially influencing patient stress levels [5,6]. However, the impact of these differences on physiological stress markers remains unclear.

Salivary biomarkers provide a non-invasive and convenient method to assess pain and stress, avoiding the drawbacks of blood sampling, such as invasiveness and patient discomfort. Key biomarkers include salivary alpha-amylase (sAA) as an indicator of sympathetic nervous system activation, and stress and cortisol levels in the hypothalamic-pituitary-adrenal (HPA) axis response [7]. Elevated sAA and cortisol levels have been correlated with perioperative anxiety and pain intensity, whereas reduced antioxidant marker levels reflect tissue damage [8]. These biomarkers offer real-time insights into patient responses and facilitate personalized interventions.

Despite these advances, there is a lack of literature that directly compares the modulation of salivary biomarkers by different local anesthetics during impacted third molar extraction. This gap underscores the need for prospective research to elucidate anesthetic-specific effects on pain and stress pathways. This prospective observational study aimed to evaluate the influence of different local anesthetics on salivary biomarkers of pain and stress in patients undergoing surgical extraction of impacted mandibular third molars. The specific objective was to measure and compare the baseline and postoperative levels of key salivary biomarkers, such as cortisol and sAA, across groups receiving 2% lidocaine and 4% articaine.

## Materials and methods

This prospective observational study adopted a non-randomized design, with patients sequentially allocated to anesthetic groups based on routine clinical decisions in the outpatient department (OPD) of the Department of Oral and Maxillofacial Surgery at the Jawahar Medical Foundation's Annasaheb Chudaman Patil Memorial Dental College, India. This approach reflects real-world practice, as allocation depends on the anesthetic stocked or preferred by the operating surgeon on the day of consultation, with surgeons encouraged to alternate anesthetics systematically with similar impaction types to promote group balance and minimize bias from surgical complexity. No trial registration was required owing to the observational nature of the study, which spanned 12 months (January 1, 2022, to December 31, 2022).

Ethical clearance was obtained from the Institutional Ethics Committee (JMF's ACPMDC/2021/IEC/SS21), and adhered to the Declaration of Helsinki. Written informed consent was obtained from all participants, and the observational design, minimal risks (such as salivary collection discomfort), and benefits (such as advancing pain management knowledge) of the study were explained. Data were anonymized to ensure confidentiality.

Eligible patients were males and females aged 18-40 years, classified as American Society of Anesthesiologists (ASA) I or II, with mesioangularly impacted mandibular third molars (Pell-Gregory Class II, Winter’s B), requiring surgical extraction under local anesthesia to standardize impaction type and reduce bias from varying surgical complexity [9]. Inclusion was further restricted to patients with minimal or no preoperative pain (Visual Analog Scale (VAS) score <10 mm) and no active infection ( excluding acute pericoronitis) to standardize baseline pain levels and prevent confounding of biomarker measurements [10]. Preoperative anxiety was standardized by enrolling only those with mild to moderate levels (Modified Dental Anxiety Scale (MDAS) scores 5-14) [11]. The exclusion criteria were allergies to amide anesthetics or epinephrine, subjects with conditions of altered cortisol secretion (Addison’s disease, Cushing’s syndrome, and congenital adrenal hyperplasia), and altered secretion of saliva (caused by atropine, calcium channel blockers, antidepressants, antihistamines, Sjogren’s syndrome, and radiotherapy). Systemic conditions (such as uncontrolled diabetes and cardiovascular disease), pregnancy, lactation, recent antibiotic/anti-inflammatory use (<two weeks), tobacco/alcohol dependence, or inability to provide consent.

The sample size was estimated a priori using an independent t-test model with the G*Power software (version 3.1.9.7; Heinrich-Heine-Universität Düsseldorf, Germany). Based on an effect size of 0.92 from a prior study comparing articaine (1.07 ± 0.25) and lignocaine (1.53 ± 0.64) on pain VAS scores, with an alpha error of 0.05 and a power of 90%, the calculation indicated a requirement of 20 patients per group (total N = 40) [12].

Patients were screened using orthopantomogram radiographs to confirm impaction severity and ensure homogeneity across groups. The baseline assessments included demographic data (age, sex, and medical history). Anxiety was assessed using the MDAS, a validated five-item questionnaire (scores 5-25) that evaluates dental anxiety through questions about anticipation, treatment, and specific procedures (such as local anesthesia) [11]. Participants completed the MDAS in a quiet consultation room before surgery, with scores categorized as mild (5-9), moderate (10-14), or high (≥15) to standardize mild to moderate anxiety (MDAS 5-14) according to the inclusion criteria. This ensured that the baseline stress biomarkers were not skewed by the extreme anxiety levels. The MDAS is considered a public domain tool that is free to use in both clinical practice and research without requiring permission or licensing fees.

Pain was evaluated using a 100-mm VAS, where patients marked their perceived pain intensity (0 = no pain, 100 = worst imaginable pain) on a horizontal line [10]. VAS was administered preoperatively to confirm minimal or no pain (VAS <10 mm), excluding patients with significant pain from conditions such as pericoronitis, which could elevate the baseline biomarker levels. The VAS is considered a public domain tool that is free to use in both clinical practice and research, without requiring permission or licensing fees.

For unstimulated saliva collection, a standardized protocol was followed to ensure sample quality and minimize contamination that could affect biomarker assays. Patients were instructed to avoid food, drink, chew gum, and perform oral hygiene activities (such as brushing and mouthwash) for at least 1 h prior to collection to prevent interference with salivary cortisol and sAA levels. This restriction minimizes dietary residues or chemical contaminants that can alter enzymatic or immunological assays. Additionally, patients were asked to rinse their mouth thoroughly with plain water (approximately 10 mL) 10 min before collection to remove debris and reduce salivary viscosity, ensuring a clean sample without stimulating saliva flow. Saliva was collected via passive drool into sterile polypropylene tubes (5 mL capacity) for 5-10 min, with patients seated comfortably and their heads tilted forward to allow saliva to pool naturally in the mouth. Collection occurred between 8-10 AM to control for diurnal variations in cortisol and sAA, which peaked in the morning and declined throughout the day. Immediately after collection, the samples were centrifuged (3000 rpm, 10 min, 4°C) to remove debris, and the supernatant was aliquoted and stored at -80°C until analysis by enzyme-linked immunosorbent assay (ELISA kit, Salimetrics, State College, USA) for cortisol and a kinetic colorimetric assay for sAA (Salimetrics, State College, USA).

Surgical extractions were performed by experienced surgeons (≥5 years) using a standardized buccal-vestibular flap with osteotomy and sectioning, as needed, under aseptic conditions in the minor operating theater. Local anesthesia was administered via inferior alveolar nerve block (IANB) with long buccal infiltration using: Group A (n = 20), 2% lignocaine hydrochloride with epinephrine 1:100,000 (Xylocaine® Injectable, AstraZeneca, UK; 3.6 mL maximum, 72 mg lignocaine); Group B (n = 20), 4% articaine hydrochloride with epinephrine 1:100,000 (Septocaine® Injectable, Septodont, France; 3.6 mL maximum, 144 mg articaine). Aspiration was confirmed by nonvascular injection. Intraoperative vital signs were monitored using a multiparameter unit (Datex-Ohmeda; GE Healthcare, USA). The conventional Halsted technique was used for the IAN block in all patients to ensure consistency. The same injection technique and anatomical landmarks were followed by all operators. Patients with a history of inhaler use were specifically excluded, as inhaled medications can alter salivary flow and affect biomarker levels.

Postoperative pain was assessed at baseline (T0), at 1-h post injection (T1), and 24-h post extraction (T24). The saliva samples were collected at the same time intervals. Two key biomarkers were analyzed, which were chosen for their established relevance to stress and pain in oral surgery. Assays were performed using a microplate reader (BioTek, USA) by a blinded technician. Surgical complexity was graded using the Pederson Index [13]. The Pederson Index is a validated scoring system designed to evaluate the anticipated surgical difficulty of extracting impacted mandibular third molars. It assesses three key parameters: angulation of the impacted tooth relative to the long axis of the second molar, depth of impaction in relation to the occlusal plane of the adjacent second molar, and the spatial relationship of the impacted tooth to the anterior border of the mandibular ramus. Each parameter was scored, and the total score (range: 3-10) was categorized as minimal (3-4), moderate (5-6), or high (7-10). This index facilitates preoperative planning by providing a standardized metric for predicting procedural challenges. Permission was obtained for using the Pederson Index.

Statistical analyses were performed using Statistical Package for Social Sciences (SPSS) software (version 26; IBM Corporation, USA). Normally distributed continuous data, as confirmed by the Shapiro-Wilk test, are presented as the mean ± SD. Baseline characteristics of the 2% lignocaine and 4% articaine groups were compared using an independent sample t-test. Changes in outcome measures within each group across the three time points were analyzed using a repeated-measures (RM) analysis of variance (ANOVA). Two-way mixed ANOVA was used to determine the interaction effect between the local anesthetic group and time. Statistical significance was set at p < 0.05, and a p-value of 0.001 was considered highly significant for all tests.

## Results

The comparison of baseline characteristics, presented in Table 1, confirmed that the 2% lignocaine and 4% articaine groups were well-matched prior to the intervention. The demographic and clinical variables, including sex distribution (p = 0.489), age (p = 0.157), and BMI (p = 0.638), were not significantly different. Critically, the key outcome measures assessed at baseline were also equivalent between the two groups. Preoperative self-reported pain (p = 0.714) and stress scores (p = 0.095), along with physiological biomarkers, such as cortisol (p = 0.301) and sAA (p = 0.606), were all comparable. The Pederson Index scores were identical between both the groups, with all patient exhibiting mesioangular impactions (score = 1), Level B depth (score = 2), and Class II ramus relationship (score = 2), resulting in a mean score of 5.0 ± 0.0 (moderate difficulty) for both groups. An independent t-test confirmed no significant differences in the surgical complexity between the groups.

**Table 1:** Comparison of baseline characteristics of the study groups p > 0.05 denotes no statistical significance using chi-square test for sex and independent t-test for age, BMI, pain, stress, Pederson Index, cortisol, and sAA levels. VAS: Visual Analog Scale; MDAS: Modified Dental Anxiety Scale; sAA: Salivary alpha-amylase

Variables	Unit	2% lignocaine (n = 20)	4% articaine (n = 20)	Test statistics	p-value
Sex	Male	8 (40%)	11 (55%)	0.65	0.489
	Female	12 (60%)	9 (45%)		
Age	Years	30.4 ± 4.81	32.2 ± 2.84	1.44	0.157
BMI	Kg/m^2^	21.1 ± 3.23	20.65 ± 2.76	0.47	0.638
Pain	VAS scores	6.75 ± 1.29	6.6 ± 1.27	0.37	0.714
Stress	MDAS scores	11.05 ± 2.35	12.35 ± 2.46	1.71	0.095
Pederson Index	Scores	5.0 ± 0.0	5.0 ± 0.0	0.0	1.00
Cortisol levels	µg/dL	0.34 ± 0.10	0.31 ± 0.13	1.05	0.301
sAA levels	Units/mL	90.05 ± 32.03	84.8 ± 31.79	0.52	0.606

The RM ANOVA results in Table 2 demonstrated a statistically significant change over time for all variables in both groups (p = 0.001). Pain and stress scores, along with cortisol and sAA levels, exhibited a consistent pattern, sharply increasing from T0 to T1, and then declining by T24. The descriptive data suggest a potential trend where the 4% articaine group may have experienced lower mean values at T1 and T24 for pain, cortisol, and sAA than the 2% lignocaine group.

**Table 2:** Comparison of both the groups for different variables at different time intervals *p = 0.001 denotes highly statistically significant values using RM ANOVA. VAS: Visual Analog Scale; MDAS: Modified Dental Anxiety Scale; sAA: Salivary alpha-amylase levels; RM: Repeated measures; ANOVA: Analysis of variance

Variables	Group	T0 (preoperative)	T1 (post injection at 1-h)	T24 (post extraction at 24-h)	F value	p-value
Pain (VAS scores)	2% lignocaine	6.75 ± 1.29	56.7 ± 10.71	27.25 ± 3.35	273.01	0.001*
	4% articaine	6.60 ± 1.27	50.85 ± 9.03	22.85 ± 5.09	338.02	0.001*
Stress (MDAS scores)	2% lignocaine	11.05 ± 2.35	17.50 ± 3.09	3.30 ± 0.73	180.12	0.001*
	4% articaine	12.35 ± 2.46	17.90 ± 2.85	2.80 ± 0.83	222.53	0.001*
Cortisol (µg/dL)	2% lignocaine	0.39 ± 0.09	1.35 ± 0.31	0.58 ± 0.13	217.90	0.001*
	4% articaine	0.31 ± 0.13	1.07 ± 0.44	0.46 ± 0.19	116.86	0.001*
sAA (units/mL)	2% lignocaine	90.05 ± 32.03	315.18 ± 112.09	135.07 ± 48.04	158.11	0.001*
	4% articaine	84.80 ± 31.79	296.8 ± 111.28	107.2 ± 31.12	142.28	0.001*

Two-way mixed ANOVA results revealed significant effects for the measured variables. For pain, a significant main effect was found for both the time (p < 0.001) and anesthetic groups (p = 0.006), but the interaction was not significant (p = 0.106), indicating that while pain levels changed significantly over time and differed overall between the groups, the pattern of change was similar for both groups. For stress, only the main effect of time was significant (p < 0.001) with no significant group differences or interactions. Crucially, for the biomarker cortisol, significant main effects of time (p < 0.001) and group (p = 0.008) were accompanied by a significant interaction (p = 0.038). Similarly, for sAA, the main effects of time (p < 0.001), group (p = 0.021), and their interactions (p = 0.047) were all significant. This indicated that the trajectory of the physiological stress response over time was significantly different between the two groups (Table 3).

**Table 3:** Interaction effects of time and group on pain, stress, cortisol, and sAA levels in mandibular third molar extractions *p < 0.05 denotes statistically significant values using two-way ANOVA VAS: Visual Analog Scale; MDAS: Modified Dental Anxiety Scale; sAA: Salivary alpha-amylase; RM: Repeated measures; ANOVA: Analysis of variance

Variables	Interaction	Mean square	F value	p-value
Pain (VAS scores)	Time	22541.18	594.53	0.001*
	Group	360.53	8.49	0.006*
	RM Factor x Group	87.76	2.31	0.106
Stress (MDAS scores)	Time	2169.63	399.71	0.001*
	Group	4.8	1.1	0.301
	RM Factor x Group	8.1	1.49	0.231
Cortisol (µg/dL)	Time	7.34	328.03	0.001*
	Group	0.17	1.1	0.008*
	RM Factor x Group	0.02	1.1	0.038*
sAA (units/mL)	Time	535019.14	300.24	0.001*
	Group	3307.5	0.27	0.021*
	RM Factor x Group	482.34	0.27	0.047*

Based on the independent t-test analysis of the change scores (T24-T0) presented in Table 4, the 4% articaine group demonstrated a statistically superior improvement across all measured outcomes compared to the 2% lignocaine group. The reduction in pain was significantly greater with articaine than for lignocaine (p = 0.008). Similarly, the reduction in stress was more pronounced in the 4% articaine group (P = 0.046). Notably, physiological biomarkers showed a significantly sharper decline in the 4% articaine group, with a greater reduction in cortisol (p = 0.009) and a substantially larger decrease in sAA levels. These results indicated that patients administered 4% articaine experienced not only significantly lower self-reported pain and stress over 24 h, but also a more rapid and pronounced reduction in their underlying physiological stress response, as evidenced by the biomarker levels. This suggests that 4% articaine is more effective than 2% lignocaine in promoting recovery after dental surgical procedures.

**Table 4:** Comparison of improvements in pain, cortisol, and sAA levels between lignocaine and articaine groups in mandibular third molar extractions (T24–T0) *p < 0.05 denotes statistically significant values using independent t-test. VAS: Visual Analog Scale; MDAS: Modified Dental Anxiety Scale; T24: Post-extraction evaluation at 24 h; T0: Preoperative evaluation; sAA: Salivary alpha-amylase

T24-T0	Group	Mean	SD	T statistics	p-value
Pain (VAS scores)	2% lignocaine	20.5	3.68	2.79	0.008*
	4% articaine	16.25	5.72		
Stress (MDAS scores)	2% lignocaine	-7.75	2.59	2.06	0.046*
	4% articaine	-9.55	2.93		
Cortisol levels (µg/dL)	2% lignocaine	0.24	0.13	2.74	0.009*
	4% articaine	0.15	0.06		
sAA levels (units/mL)	2% lignocaine	45.03	16.01	5.51	0.001*
	4% articaine	22.63	8.6		

## Discussion

The results of this prospective observational study provided evidence that 4% articaine outperformed 2% lignocaine in reducing postoperative pain and physiological stress responses during surgical extraction of impacted mandibular third molars. The findings of this study align with the hypothesis that the pharmacological properties of articaine, such as its rapid onset and enhanced tissue penetration, contribute to better pain control and stress attenuation than lignocaine [14].

The superior performance of 4% articaine in mitigating pain and stress biomarkers (cortisol and sAA) is consistent with established pharmacological advantages. The thiophene ring of articaine enhances lipid solubility, allowing better diffusion through the bone and soft tissues, which is particularly beneficial in mandibular third molar extractions that require deep anesthesia via IANB. Studies have shown that articaine achieves anesthesia faster (1-3 min vs. lignocaine’s 2-5 min) and maintains it longer, reducing intraoperative discomfort and the need for supplemental injections [13-15]. This likely explains the lower pain and biomarker levels in the articaine group, as effective anesthesia minimizes noxious stimuli that trigger sympathetic nervous system activation (reflected by sAA) and HPA axis responses (reflected by cortisol). Previous research supports this finding, noting that reduced pain perception correlates with lower sAA and cortisol levels in dental procedures, as these biomarkers are sensitive to acute stress and tissue trauma [16,17].

The emergence of fear, anxiety, and stress in adult populations undergoing surgical extraction of impacted mandibular third molars is attributed to the physiological activation of the sympathetic nervous system, particularly the sympathetic adrenal medullary system and the HPA axis [17]. Cortisol and sAA are reliable biomarkers associated with stress and serve as indicators of sympathetic activation and HPA axis functionality [18]. Given that these biomolecules are present at physiological concentrations in the saliva, they can be effectively quantified using salivary collection methods. The non-invasive nature of saliva collection is not anticipated to exacerbate stress levels in the individuals being assessed [17,18]. Furthermore, saliva collection offers a straightforward, hygienic, and economically viable approach for diagnostic evaluation.

The significant group and interaction effects for cortisol and sAA suggested that articaine not only reduced peak stress responses but also facilitated a faster return to baseline levels. This may be attributed to the ester linkage of articaine, which allows rapid plasma hydrolysis, potentially minimizing systemic stress compared to the amide structure of lignocaine [14]. A comparative study on third molar surgery found that articaine provided superior postoperative analgesia, reducing VAS scores and inflammatory markers, which aligns with our findings [19]. Additionally, the higher potency (1.5-2 times that of lignocaine) likely contributed to deeper anesthesia, dampening stress pathways more effectively. This study’s novel focus on salivary biomarkers addresses a literature gap, as none of the studies have directly compared the anesthetic effects on physiological stress markers in this context.

However, contrasting results were reported in a study on dental treatment in pediatric patients, where it was found that both 4% articaine and 2% lignocaine showed comparable reduction in pain scores [20]. This discrepancy likely arose from methodological differences such as non-standardized impactions or the use of buffered anesthetics, which can alter the onset and duration. Our study’s strict standardization (such as identical Pederson scores, VAS <10 mm, and MDAS 5-14) minimized such confounders, allowing clearer detection of the benefits of articaine. Furthermore, the focus on biomarkers such as cortisol and sAA, rather than clinical outcomes alone, provided a novel perspective, as prior studies did not examine physiological stress responses in this context.

The clinical implications of these findings are significant. Superior pain control and stress reduction with 4% articaine suggest its preferential use in mandibular third molar extractions, particularly in patients with mild to moderate anxiety (MDAS 5-14), where stress amplification is a concern. Reduced biomarker levels may correlate with fewer postoperative complications such as swelling or delayed healing, enhancing patient recovery and satisfaction. Salivary biomarker monitoring could be integrated into clinical practice to tailor anesthesia choices, especially in high-stress or complex cases, offering a non-invasive method for assessing patient responses. The rapid onset and prolonged effect of articaine may also reduce reliance on postoperative analgesics, thereby addressing concerns regarding opioid use in dental surgery.

The limitations of the study include its non-randomized, observational design, which may introduce allocation bias despite systematic efforts to alternate anesthetics. The small sample size (n = 20 per group) limits the generalizability, particularly to diverse populations or more complex impacts. The 24-h follow-up period may miss longer-term differences in recovery or complications. The single-center setting may not reflect broader clinical practices, and reliance on self-reported measures (VAS and MDAS) may introduce subjectivity. Excluding patients with conditions such as Sjogren’s syndrome or altered cortisol secretion enhances internal validity but restricts applicability to such populations. The focus on only two biomarkers (cortisol and sAA) excludes other potential markers such as immunoglobulin A (IgA), which could provide additional insights into inflammation. Despite being non-invasive and easy to collect, salivary biomarkers have limitations such as variability due to flow rate, circadian rhythm, hydration, and oral health status. Contamination and inter-individual differences may affect accuracy, and salivary levels may not always precisely reflect systemic changes. Moreover, we did not include extraction of impacted maxillary third molars. Future research should employ randomized controlled designs, larger and more diverse cohorts, extended follow-up periods, and additional biomarkers to validate these findings and explore long-term outcomes.

## Conclusions

This prospective observational study demonstrated that 4% articaine outperformed 2% lignocaine in reducing postoperative pain and physiological stress during surgical extraction of mesioangular-impacted mandibular third molars. The superior efficacy of 4% articaine compared to 2% lignocaine is likely due to its ability to modulate salivary cortisol and sAA more effectively, leading to reduced physiological stress responses. These findings advocate the preferential use of articaine in such procedures, highlighting the importance of salivary biomarker monitoring to personalize anesthesia and improve patient outcomes.
